# The Cardiovascular Effects of Cocoa Polyphenols—An Overview

**DOI:** 10.3390/diseases4040039

**Published:** 2016-12-17

**Authors:** Ana Clara Aprotosoaie, Anca Miron, Adriana Trifan, Vlad Simon Luca, Irina-Iuliana Costache

**Affiliations:** 1 Department of Pharmacognosy, Faculty of Pharmacy, University of Medicine and Pharmacy Grigore T. Popa Iasi, Universitatii Str. 16, 700115 Iasi, Romania; anca.miron@umfiasi.ro (A.M.); adriana_trifan@yahoo.com (A.T.); simon-vlad.v.luca@d.umfiasi.ro (V.S.L.); 2 Department of Cardiology, Faculty of Medicine, University of Medicine and Pharmacy Grigore T. Popa Iasi, Universitatii Str. 16, 700115 Iasi, Romania; irinaiulianacostache@yahoo.com

**Keywords:** cocoa, flavanols, proanthocyanidins, cardiovascular health

## Abstract

Cocoa is a rich source of high-quality antioxidant polyphenols. They comprise mainly catechins (29%–38% of total polyphenols), anthocyanins (4% of total polyphenols) and proanthocyanidins (58%–65% of total polyphenols). A growing body of experimental and epidemiological evidence highlights that the intake of cocoa polyphenols may reduce the risk of cardiovascular events. Beyond antioxidant properties, cocoa polyphenols exert blood pressure lowering activity, antiplatelet, anti-inflammatory, metabolic and anti-atherosclerotic effects, and also improve endothelial function. This paper reviews the role of cocoa polyphenols in cardiovascular protection, with a special focus on mechanisms of action, clinical relevance and correlation between antioxidant activity and cardiovascular health.

## 1. Introduction

Cardiovascular diseases (CVDs) are the leading cause of mortality and morbidity worldwide. Every year, about 17 million people die from CVDs, which represent almost one third of all deaths in the world [1]. By 2030, it is estimated that about 23.3 million people will die due to CVDs [2]. The major heart health issues are: high blood pressure (hypertension), heart ischemic and cerebrovascular diseases. Hypertension is the main risk factor for CVDs. It is a silent killer that affects one third of the worldwide adult population and contributes to nearly 45% of heart ischemic diseases and 51% of stroke cases around the globe [3]. It is considered that every 20-mmHg drop in systolic blood pressure (SBP) would reduce the relative risk of ischemic heart disease mortality by 50% [2]. Other significant behavioural risk factors of CVDs include: unhealthy diet, physical inactivity, smoking, alcohol, exposure to continuous stress, diabetes, obesity, hyperlipidemia. Change of lifestyle and diet is considered to be one of the main tools for the prevention of CVDs, particularly ischemic heart diseases, atherosclerosis, and arterial thrombosis. Epidemiological studies have shown that a diet rich in fruits and vegetables with a high intake of polyphenols have beneficial effects on human health; it can protect and limit the incidence of CVDs, cancer or age-related degenerative diseases. Cocoa is one of the most polyphenol-rich foods that is widely consumed in the world. In recent years it has received much attention due to its antioxidant, cardioprotective, neuroprotective, and chemopreventive properties [4]. 

In this review, we aim to describe the current evidence on the cardioprotective effects of cocoa polyphenols and cocoa derived products with a particular focus on the main mechanisms of action, clinical relevance, and the link between antioxidant activity and cardiovascular health. The limitations of these studies and future perspectives on cocoa polyphenols research are also presented.

## 2. Polyphenols Composition of Cocoa

**Figure 1:**
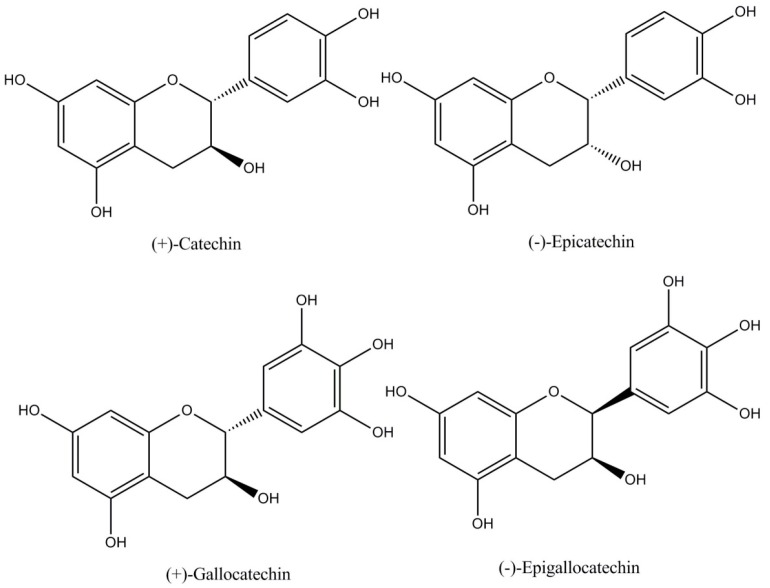
Cocoa flavanols.

**Figure 2:**
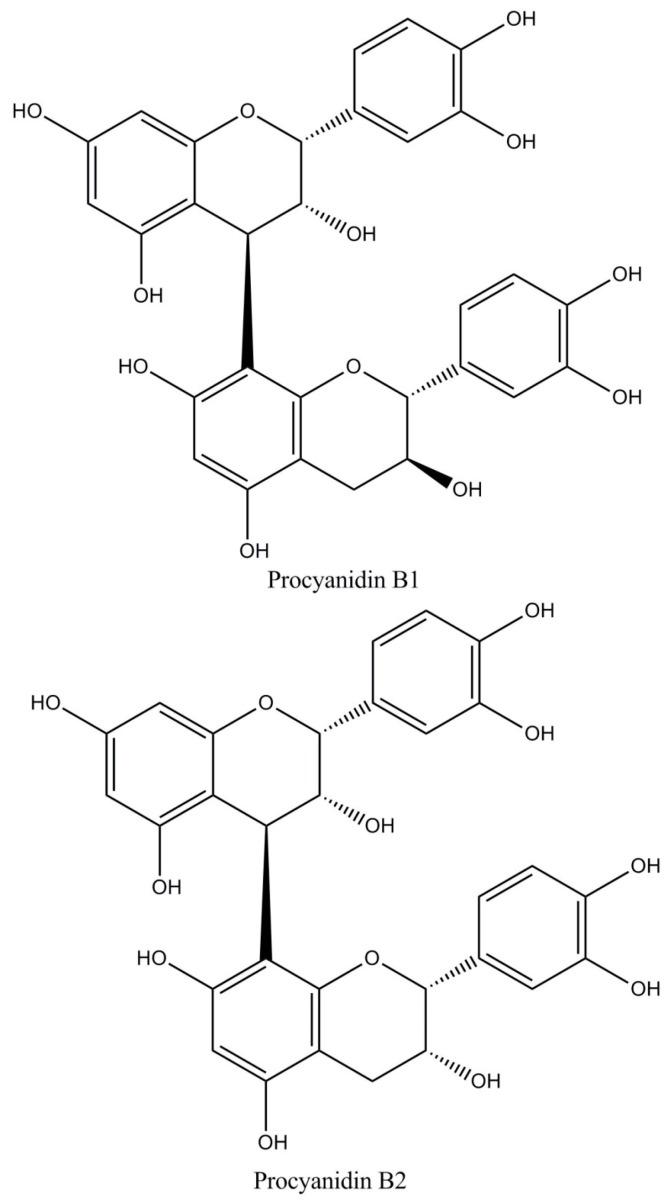
Major proanthocyanidins-dimers in cocoa.

**Figure 3:**
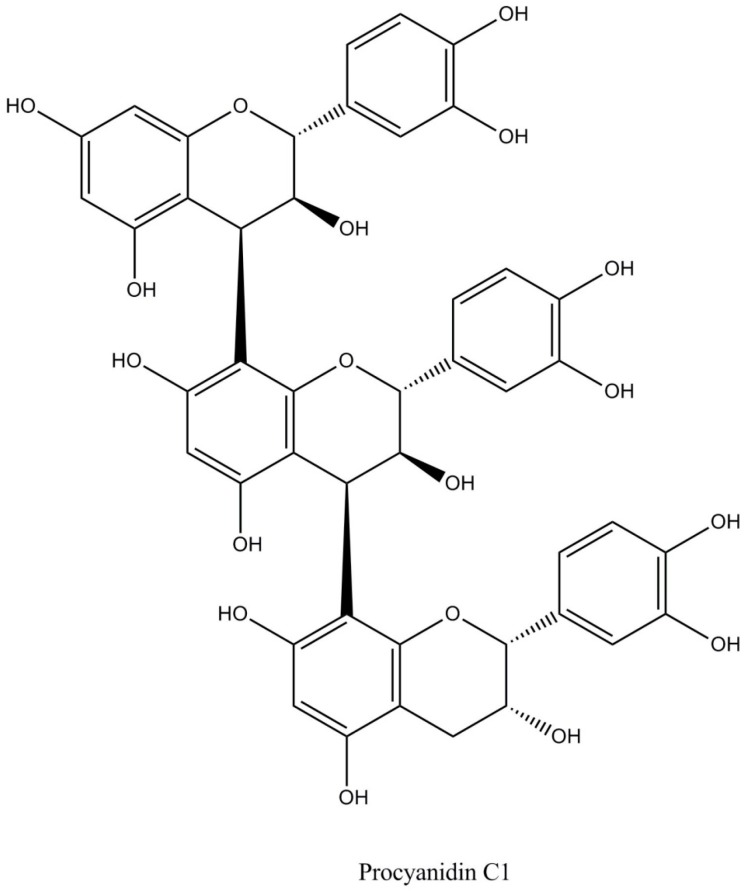
Proanthocyanidins-trimers in cocoa.

Raw dried cocoa beans (*Theobroma cacao*, Malvaceae, cacao tree) contain high levels of polyphenols (12%–18% of whole beans dry weight). In addition, other important bioactive phytochemicals of cocoa beans are methylxanthines (4%) with theobromine as the main compound [4,5]. The main categories of cocoa polyphenols are:

(i) Flavan-3-ols or catechins (29%–38% of total polyphenols) as monomers and polymers. The monomers include (−)-epicatechin, (+)-catechin, (+)-gallocatechin, and (−)-epigallocatechin (Figure 1). (−)-Epicatechin constitutes up to 35% of total polyphenols being the most abundant polyphenol both in cocoa and cocoa derived products (chocolate) [4,6]. Flavanols are commonly found in cocoa as R-forms [7].
(ii) Proanthocyanidins (58%–65% of total polyphenols) are polymers of epicatechin and catechin molecules linked by 4→8 and 4→6 bonds. In cocoa, mainly oligomeric proanthocyanidins (degree of polymerization = 10) are found, and dimers to hexamers predominate. The most important cocoa proathocyanidins are dimers: B1 [epicatechin-(4β→8)-catechin], B2 [epicatechin-(4β→8)-epicatechin] (Figure 2), B3 [catechin-(4α→8)-catechin], B4 [catechin-(4α→8)-epicatechin], B5 [epicatechin-(4β→6)-epicatechin], and trimers: C1 [epicatechin-(4β→8)_2_-epicatehin] (Figure 3) [4].
(iii) Anthocyanins (4% of total polyphenols), such as: cyanidin-3α-l-arabinoside, cyanidin-3 β-d-galactoside [8], and leucoanthocyanins (L1, L2, L3, L4) [4].

Other polyphenols from cocoa are: flavones (apigenin, quercetin, luteolin, kaempferol and their glycosides), polyphenolic acids (caffeic acid, chlorogenic acid, ferulic acid, coumaric acid, and syringic acid), caffeoyl-conjugates (clovamide, deoxyclovamide), stilbens (trans-resveratrol and its glycoside, trans-piceid) [4,5]. The polyphenols profile in cocoa varies depending of several factors, such as: plant genotype, geographic area, ripeness degree of beans, cocoa processing [4]. Alongside with ground cocoa, green tea and wine, dark chocolate contains high amounts of polyphenols (460–610 mg/kg) [5,9].

## 3. Bioavailability of Cocoa Polyphenols

**Figure 4:**
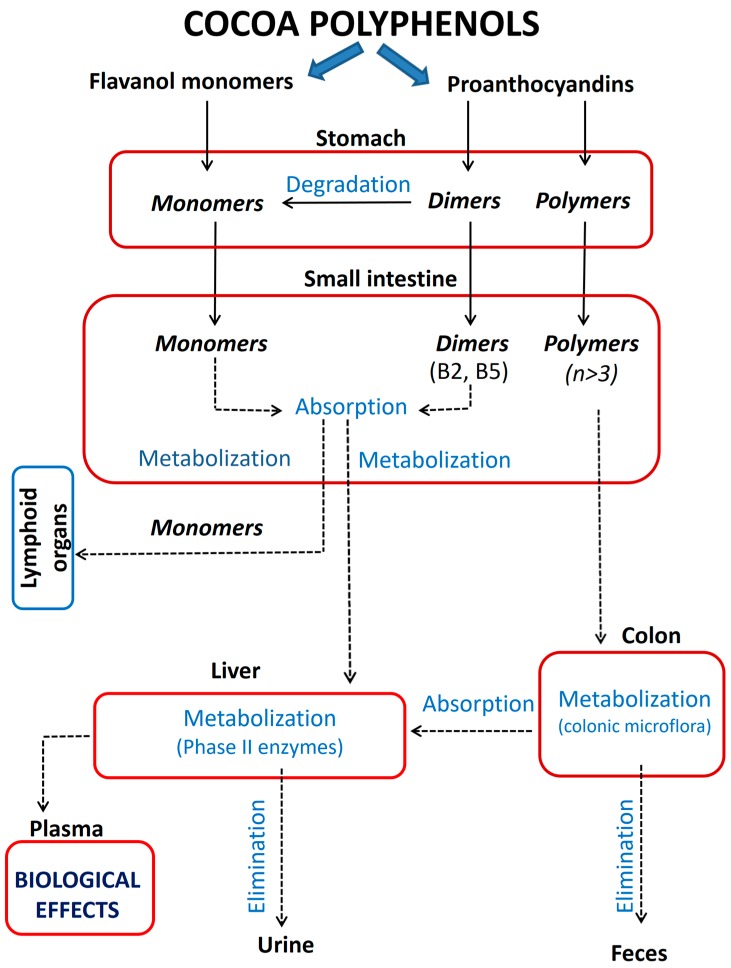
Pharmacokinetic profile of cocoa polyphenols.

Cocoa polyphenols have a relatively low bioavailability. They achieve low plasma Cmax, have a short half-life and present a rapid excretion [10]. The bioavailability is strongly influenced by molecular size; thus, compounds with low molecular weight like flavanol monomers can achieve higher concentrations in the blood and can reach the target organs in the body [10,11]. Monomers and dimeric or trimeric proanthocyanidins are quite stable in the gastric environment and they can travel to the small intestine where they could be absorbed (Figure 4) [6]. Monomers are able to cross the gut barrier and are absorbed, being largely distributed in the lymphoid organs (thymus, spleen, mesenteric lymphoid nodes), liver, or testes [12]. At two to three hours after ingestion of cocoa products, flavanols achieve the peak concentrations in the plasma and these are typically in the nanomolar or low micromolar range, depending on the administered dosage [8,12]. (−)-Epicatechin shows a higher bioavailability than other cocoa flavanols (catechins, galloylated derivatives) [13]. After the intake of a cocoa beverage that contains 323 mg of flavanols, the plasma epicatechin concentration was >10% of the peak plasma catechin level (5.92 μM compared to 0.16 μM) [14]. The different stereochemistry of epicatechin could allow a greater intestinal permeation of this flavanol [2]. Dimeric and trimeric proanthocyanidins are poorly absorbed through gastrointestinal tract (about 10–100 times less compared to epicatechin and catechin) [2]. In the acidic conditions of stomach some dimers can be degraded to epicatechin and undergo interflavan bond conversion. After the intake of cocoa with large amounts of flavanol monomers and dimers, the occurrence of B2 and B5 dimers in human plasma has been reported but in modest concentrations (41 nM and 55 nM, respectively) [14]. Oligomeric proanthocyanidins with degree of polymerization higher than three and polymeric flavanols are not absorbed in native forms [15]. In the colon, they are metabolized by the local microbiota within 48 h into various low-molecular weight phenolic compounds, such as hydroxyphenylvalerolactones, hydroxyphenylpropionic acids, hydroxyphenylacetic acids, hydroxycinnamic acids, and hydroxybenzoic acids [14,15]. These metabolites are more available, can be absorbed and reach the liver, and some of them show significant biological properties [12,15]. The mammalian metabolism of simple cocoa flavanols occurs in the liver and small intestine through glucuronidation, sulfation, and *O*-methylation [16], and the circulating flavanols are predominantly the conjugated forms [2]. The major metabolite of epicatechin in the plasma is 4’-*O*-methyl-epicatechin-7β-d-glucuronide [17]. Also, flavonoid metabolism can occur in the vascular endothelial cells. In human umbilical vein endothelial cells (HUVEC), (−)-epicatechin is converted via catechol-*O*-methyltransferase (COMT) to 3’-*O*-methyl-epicatechin and 4’-*O*-methyl-epicatechin, metabolites with physiological relevance. Although the endothelial concentrations of epicatechin are not known so far, it is appreciated that they are higher than the plasma concentrations [17]. Flavanol monomers have short-half lives in the body and are rapidly excreted in the urine or bile as conjugates [14]. After ingestion of 40 g chocolate bars, there has been reported a half-live of 1.9 h for (−)-epicatechin [2]. Some of flavanol metabolites such as glucuronides are no longer considered only excretion products. They appear to play an important role as transport metabolites in plasma to target cells [17]. The chirality of flavanol molecules and also cocoa-containing matrices may affect the bioavailability of polyphenols. (+)-Catechin enantiomer from cocoa beans is more bioavailable than (−)-catechin form, which is predominantly present in the commercial chocolates. Simultaneous use of carbohydrates increases significantly the absorption of polyphenols [8], while the lipids in cocoa matrix seem to stimulate the digestion of flavanols in the duodenum [2]. Conflicting data on the influence of milk on cocoa polyphenols bioavailability have been reported [10]. It has been suggested that milk proteins bind cocoa polyphenols, which causes a decrease of polyphenols absorption in the gastrointestinal tract, but this hypothesis has not been confirmed for (−)-epicatechin when cocoa was consumed with milk [10].

## 4. Cardioprotective Effects of Cocoa Polyphenols

**Figure 5:**
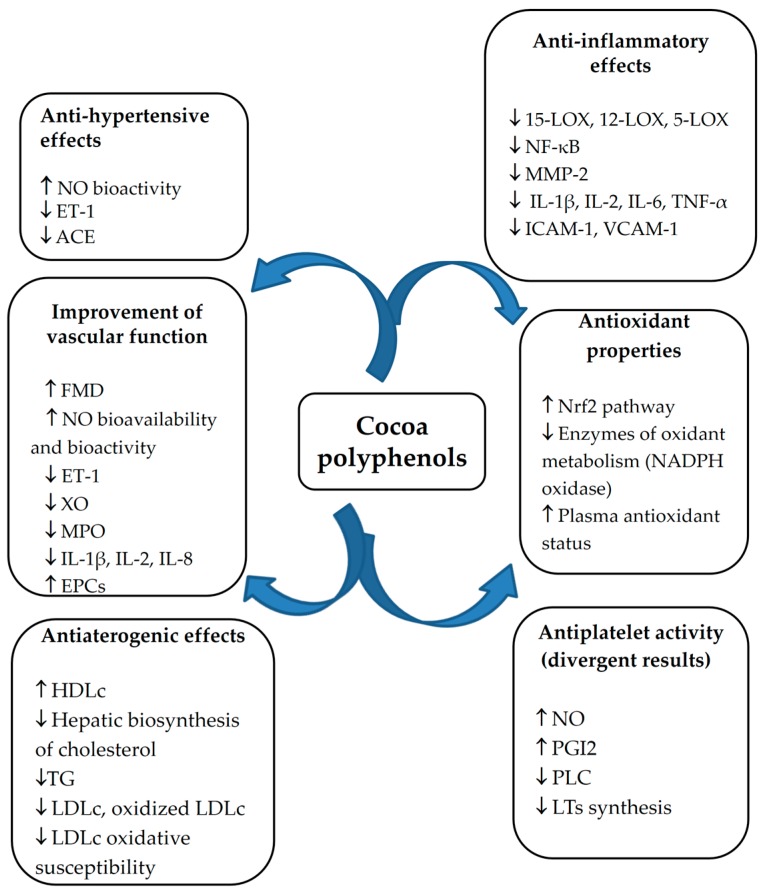
Main cardioprotective properties of cocoa polyphenols. Abbreviations: ACE, angiotensin-converting enzyme; EPCs, endothelial progenitor cells; ET-1, endothelin 1; FMD, flow-mediated dilation; HDLc, high-density lipoprotein-cholesterol; ICAM, intercellular adhesion molecule; IL, interleukin; LDLc, low-density lipoprotein-cholesterol; LOX, lipooxygenase; LTs, leukotrienes; MMP-2, matrix metalloproteinase 2; MPO, myeloperoxidase; NADPH, reduced nicotinamide adenine dinucleotide phosphate; NF-κB, nuclear factor kappa-light-chain-enhancer of activated B cells; NO, nitric oxide; Nrf2, nuclear factor erythroid-related factor 2; PGI2, prostaglandin I2; PLC, phospholipase C; TG, triglycerides; TNF, tumor necrosis factor; VCAM, vascular cell adhesion molecule; XO, xanthine oxidase.

Numerous epidemiological studies, short-term human intervention studies, or small-cohort studies, showed that the intake of cocoa and polyphenols-rich cocoa products is correlated with cardiovascular benefits in humans [16]. Cocoa flavanols and proanthocyanidins are pleiotropic molecules that can influence various biomedical markers and clinical endpoints of cardiovascular health. They improve cardiovascular function but also facilitate endogenous repair mechanisms [18]. Experimental research and clinical trials have investigated mainly the effects of cocoa products and cocoa polyphenols on oxidative stress and plasma antioxidant capacity, endothelium-dependent vasomotor function and arterial flow mediated dilatation (FMD), nitric oxide (NO) metabolism and activity, blood pressure, platelet function, lipid profile, and vascular inflammation [5,10]. An overview of the most recent clinical trials (2014–2016) with cocoa flavanols is shown in Table 1. The potential cardioprotective properties of cocoa include mainly antihypertensive, anti-atherogenic, and anti-inflammatory activities as well as inhibition of the platelet activation and aggregation, and attenuation of endothelial dysfunction (Figure 5).

### 4.1. Antioxidant Activity

Oxidative stress is actively involved in CVDs pathogenesis. The increase of reactive oxygen species (ROS) production, mainly of superoxide anion radical, affects NO metabolism and facilitates endothelial dysfunction [45]. Besides, lipid peroxidation contributes essentially to the initiation and progression of atherosclerosis. Cocoa is a rich source of antioxidants. The assessment of the antioxidant capacity of 1113 different foods showed that of 50 products exhibiting the highest activity, five products are cocoa-based [12]. Cocoa powder has a higher antioxidant activity than green tea (1000 ORAC units compared to 800 ORAC units), and the content of total polyphenols is positively correlated with ORAC value [14]. Cocoa polyphenols mainly flavanols and oligomeric proanthocyanidins have shown significant in vitro antioxidant properties. They act as scavengers of various radicals (DPPH, ABTS, superoxide anion, peroxynitrite, hypochloride), inhibit lipid peroxidation and chelate pro-oxidants metal ions (Fe^2+^) [4,46]. The main structural features of polyphenols that are responsible for their ROS quencher activity are catechol groups, phenolic quinoid tautomerism and delocalization of electrons [14]. Epicatechin enhances plasma antioxidant capacity and protects erythrocyte membrane from lipid peroxidation. Also, flavanol monomers and oligomeric proanthocyanidins protect against in vitro erythrocyte hemolysis induced by 2,2’-azobis (amidopropane)-dihydrochloride (AAHP) [14]. In animal experimental models, long-term feeding studies with flavanol-rich cocoa products showed an increase in total plasma antioxidant capacity [14]. In rat brain homogenates, cocoa polyphenolic extracts inhibit lipid peroxidation acting as chain-breaking antioxidants [11]. The suppressive effect of cocoa flavanols on lipid peroxidation has been suggested by some studies, which showed a decrease of the plasma level of F2 isoprostanes. However, many in vivo investigations did not identify similar results [17]. Also, some studies in healthy human subjects did not reveal changes of oxidative stress biomarkers consecutive to cocoa consumption. It is possible that the antioxidant effects of cocoa products could be better expressed in pathological conditions [4]. At the same time, it is difficult to translate in vivo antioxidant activity of cocoa polyphenols. The poor bioavailability and low plasma concentrations of cocoa polyphenols do not support a direct antioxidant activity. It is more likely that at low physiological levels reached by flavanols, the latter act indirectly as antioxidants by a specific interaction with lipids and enzymes associated with oxidant metabolism and cardiovascular diseases (NADPH oxidases, lipoxygenases, myeloperoxidase) [6,17,47]. Besides, cocoa polyphenols upregulate antioxidant defense response, such as the nuclear factor erythroid 2-related factor 2 (Nrf2) signaling pathway, a master regulator of cellular resistance to oxidants [46]. Therefore, they can attenuate the rise of intracellular oxidants via NF-κB activation [48]. 

### 4.2. Modulation of Endothelium-Dependent Vasomotor Function

The endothelium participates critically to maintain vascular homeostasis. It plays a pivotal role in the regulation of vascular tone, vascular permeability, platelet activity and aggregation. Its effects are mediated by a complex network of molecules, such as NO, endothelin-1 (ET-1), prostacyclin, cell adhesion molecules [49], leukotrienes, prostaglandins, catecholamines, vasoactive peptides (angiotensin-II) [48]. The impairment of functional properties of endothelium is involved in the initiation and progression of atherosclerosis and other CVDs [9,47]. The undesirable phenotypic changes of endothelium, such as vasoconstriction, proliferation of arterial wall, inflammation, and thrombosis, occur with endothelial dysfunction [49]. The quantification of endothelial function in humans is accomplished by the use of brachial artery flow-mediated dilation technique (FMD). FMD value largely reflects NO-mediated arterial function (including that of coronary arteries) and low levels are associated with cardiovascular events and elevated risk factors for CVDs [47,49]. Most studies have shown that the acute or chronic (≥1 week) intake of cocoa/chocolate increases FMD value in healthy young and elderly subjects, smokers, obese, patients with coronary artery diseases and arterial hypertension, or end-stage renal diseases with chronic hemodialysis, and diabetics [21,49]. Acute ingestion of the medium and high dose of flavanols (321 mg of flavanols/dose, three doses/day) in elderly type 2 diabetic patients improves FMD value by over 40%, but the vascular response to oral treatment with nitroglycerin was not affected by the dietary intervention [50]. The intake for seven days of cocoa containing 74 mg of flavanols and 232 mg of procyanidins (three doses/day) improved significantly FMD in smokers, but after the cessation of cocoa consumption and a seven-day wash-out period, FMD level returned to preintervention values [16].

In addition, metabolites of epicatechin may have a relevant physiological activity. It was observed that the increase of FMD level correlates with plasma levels of epicatechin metabolites after cocoa ingestion [49]. Thus, FMD response follows a similar temporal pattern with the appearance of (−)-epicatechin metabolites in plasma, peak effects being observed two-hours after cocoa ingestion [17,49]. This short-time effect can be explained by the reduction of superoxide anion-mediated loss of NO and oxidative stress via inhibition of NADPH oxidase activity [17]. The *O*-methylated metabolites of (−)-epicatechin (3’-*O*-methyl epicatechin, 4’-*O*-methyl epicatechin) that occur in plasma in free or glucuronidated form significantly inhibit endothelial NADPH oxidase activity [13]. The increase of circulating bioactive NO pool (RXNO) plays an important role in the attenuation of vascular NO deficit in patients with risk cardiovascular factors and the restoration of endothelium-dependent vasodilation. In smokers, the consumption of a flavanol-rich cocoa beverage (176–185 mg/mL flavanols with 20–22 mg/mL epicatechin and 106–111 mg/mL proanthocyanidins) at 12 h after cessation of smoking and during abstinence dose-dependently increases FMD value by almost 50% and RXNO level by more than a third [51]. An improvement of endothelial function in healthy subjects was also observed after combined intake of low amounts of cocoa flavanols and nitrate-rich foods [32].

Repeated administration of high-flavanol cocoa produces a longer-term effect that is characterized by an increase in the baseline level of FMD. This effect can be mediated by changes in gene expression and protein synthesis (endothelial nitric oxide synthase, eNOS) [17]. Besides the improvement of NO bioavailability and bioactivity, the positive endothelial effects of cocoa can be correlated with other mechanisms, such as modulation of PGI2 and leukotrienes, reduction of xanthine oxidase and myeloperoxidase activities, suppression of the proinflammatory cytokines IL-1β, IL-2 and IL-8 production, inhibition of ET-1 release [52], decrease of the biomarkers associated with vascular damage like monocyte CD62L expression and the formation of elevated endothelial microparticles [30], and mobilization of functionally unaltered circulating angiogenic cells (EPCs) [18]. Also known as endothelial progenitor cells, EPCs originate from bone marrow cells and are capable to differentiate into mature endothelial cells with unaltered functional properties [53]. EPCs participate and significantly contribute to the endothelial reparative processes, neoangiogenesis tissue regeneration, and platelet function regulation via direct effects and indirect activities by production of paracrine/juxtacrine signals (proangiogenic cytokines, angiogenic growth factors) [53,54,55]. EPCs are currently considered potential biomarkers of cardiovascular risk. The number and functionality of EPCs are adversely affected in some conditions such as systemic hypertension, heart failure, coronary artery disease, stroke, atherosclerosis, diabetes mellitus, chronic kidney disease or chronic venous insufficiency, or in chronic inflammatory diseases (rheumatoid arthritis, systemic lupus erythematosus, systemic sclerosis, Kawasaki’s diseases). An increase in EPCs mobilization has been associated with acute ischemic events (acute myocardial infarction, unstable angina) [53,54,56].

Although the improvement of FMD induced by cocoa is supported by the preponderance of data, it is less clear what the minimal doses of cocoa/chocolate needed to be ingested to exert positive vascular effects are. Monahan [49] specifies that the minimal amount of cocoa that causes an increase in FMD value in healthy elderly individuals appears to be greater than 2 g and at most 5 g. Therefore, it seems that a pronounced and consistent increase in endothelial function occurs for large doses of about 900 mg of flavanols/day whereas low doses of 80 mg of flavanols/day do not produce a significant effect [34]. 

European Food Safety Authority (EFSA) [57] recommends 200 mg of cocoa polyphenols daily (provided by 2.5 g of polyphenol-rich cocoa powder or 10 g of polyphenol-rich dark chocolate) in order to obtain endothelium-dependent vasodilation in general population in the context of a balanced diet.

### 4.3. Effects on Blood Pressure

The research of cocoa effects on blood pressure (BP) was initiated by observations on Kuna Indians from the coast of the Panama islands. This population has very low levels of BP and incidence of hypertension and CVDs but the migration in the Panama urban continental areas leads to negative impact on the cardiovascular health. Subsequent investigations showed that the differences between the two populations are due mainly to the dietary habits. The high intake of flavanol-rich home-prepared cocoa in the island-dwelling Kuna in comparison to the mainland-dwelling Kuna (up to 10 times higher) appear to confer relevant beneficial effects [2,6,50]. Different epidemiological studies (Zutphen Elderly Study, Stockholm Heart Epidemiology Program) have reported a significant inverse relationship between the intake of cocoa/dark chocolate and cardiac mortality [58]. Many experimental studies and randomized intervention trials have shown BP-lowering effects of cocoa polyphenols and cocoa products. Single oral administration of flavonoid-enriched cocoa powder (128.9 mg/g of total procyanidins, 19.36 mg/g of epicatechin) at different doses (50, 100, 300, 600 mg/kg) clearly decreases BP in spontaneously hypertensive rats. A dose of 300 mg/kg cocoa powder produces the maximum effect, which is similar to that induced by captopril (50 mg/kg), a well-known antihypertensive drug [59]. Also, the presence of epicatehin in the diet of Sprague-Dawley rats (0.4 g epicatechin/100 g diet) for eight days prevents the increase of BP induced by NO deficiency associated to the NO-synthase inhibitor, nitro-l-arginine methyl ester (l-NAME) pretreatment [6]. It seems that epicatechin exerts the antihypertensive effects only in a pathological state. Besides, the epicatechin treatment limits infarct size in animal model of myocardial ischemia-reperfusion injury and after permanent coronary occlusion [60]. In humans, the BP-lowering properties of cocoa and cocoa polyphenols were assessed in studies with diverse design: various groups of subjects (young, elderly, overweight, obese, hypercholesterolemic, pre-hypertensive, hypertensive adults), different cocoa products and flavanols doses, and various duration of administration. A meta-analysis of 20 randomized controlled trials, which evaluated the cocoa polyphenols effects on BP, reported a small and significant reduction in both systolic blood pressure (SBP; −2.77 mmHg) and diastolic blood pressure (DBP; −2.20 mmHg) [2,61]. Other meta-analyses have identified large drops in both SBP and DBP: Desch 2010 (−4.52 mmHg SBP; −2.50 mmHg DSP) [62], Taubert 2007(−7.6 mmHg SBP; −4.8 mmHg DSB) [63] or Grassi 2005 (−11.30 mmHg SBP; −7.60 mmHg DSB) [2,64]. The study design but mostly the intake of various doses of cocoa flavanols (30–1080 mg) could explain this large variation of BP values. 

Daily consumption of cocoa beverages containing high-flavanol (990 mg), intermediate-flavanol (520 mg) and low-flavanol (45 mg) in the elderly subjects, for 8 weeks, produces a significant reduction in SBP and DSP only for high and intermediate doses of flavanols [2,65]. The intake of cocoa containing 1052 mg of flavanols decreased significantly BP (−5.3 mmHg SBP; −3 mmHg DSP) in patients with untreated moderate hypertension [58].

The meta-analysis by Ried [61] revealed that the BP-lowering effects of cocoa were more pronounced in younger subjects, hypertensive patients, and in the studies with flavanol-free controls. Also, the change of BP was more significant in studies lasting more than two weeks. It seems that the high-flavanol cocoa produces a sustained reduction in patients with hypertension when treatment lasts for at least seven days, which suggests a time-dependent effect [60]. On the other hand, Hooper et al. [66] mentioned that the BP lowering effects of cocoa and chocolate appeared greater in studies with higher doses and shorter duration. These discrepancies could be due to differences in the backgrounds of cohorts and cocoa type products or absence of suitable placebos [52]. The amount of sugar associated with cocoa products may influence the BP lowering effects. Cocoa products with more than 10 g of sugar cause smaller reduction in SBP and DBP (−1.32 mmHg) in comparison with products containing less than 10 g of sugar (−2.52 mmHg SBP; −2.35 mmHg DBP) [2]. Vlachopoulos et al. [67] have found that high habitual cocoa consumption (≥4.63 g/day) decreases aortic stiffness and wave reflections that have a causative role in the pathogenesis of systolic hypertension. 

The positive effects of cocoa on BP are supported mainly by the increase of endothelial bioavailability and bioactivity of NO and the inhibition of endothelial vasoconstriction. Up-regulation of NO is achieved by several mechanisms, such as: (i) the augmentation of eNOS activity, enzyme, which facilitates the production of NO from L-arginine. Cocoa flavanols such as epicatechin acutely enhances NO concentrations in rabbit aortic rings and in cultured human coronary arteries [2]; (ii) down-regulation of NADPH-oxidase and the reduction of superoxide anion and cellular oxidants levels. Superoxide anion is primarily generated from the electron transport chain but also through NADPH oxidase, xanthine oxidase and cytochrome P450 activities. It modulates NO availability in the smooth muscle cells. Superoxide anion directly reacts with NO producing peroxynitrite that enhances local oxidative stress. Cocoa flavanols prevent NO loss via superoxide anion reaction. In addition, they protect against the oxidative loss of tetrahydrobiopterin and prevent dysfunctional production of superoxide anion via eNOS uncoupling [6]; (iii) inhibition of arginase activity and the preservation of the intracellular arginine pool, which is the substrate for NO synthesis [6]. Epicatechin inhibits arginase-2 mRNA expression and activity in human umbilical vein endothelial cells [8], and flavanols-rich cocoa decreases the arginase activity in rat kidney and human erythrocytes [6]. Other anti-hypertensive mechanisms of cocoa polyphenols include: (i) inhibition of endothelin-1 (ET-1), an endothelium-derived peptide with vasoconstrictive effect. At low micromolar concentrations, cocoa flavanols inhibit transcription of ET-1 gene in endothelial cells andprevent ET-1 ROS production [2]; (ii) modulation of renin-angiotensin-aldosteron system. Cocoa flavanols and proanthocyanidins inhibit the renal angiotensin-converting enzyme (ACE) activity, which is involved in the regulation of BP by transforming angiotensin I in angiotensin II, a potent vasoactive and inflammatory peptide. In addition, cocoa polyphenols reduce the prooxidant effects of angiotensin II [58]; (iii) improvement of sympathovagal balance [34].

### 4.4. Antiplatelet Effects

Platelets activation and aggregation have a crucial importance in the ethiology and pathogenesis of CDVs and cerebrovascular diseases. Several studies reported that the intake of cocoa and dark chocolate in moderate amounts acutely or chronically determines a significant inhibition of platelet aggregation and adhesion in healthy volunteers, smokers or people who suffered a heart transplant [9]. The consumption of flavanol-rich cocoa beverages (600 and 900 mg of flavanols) causes a significant inhibition of platelet activation and aggregation, and inhibits platelet-monocyte conjugate and platelet-neutrophil conjugate formation [68]. The administration of high (897 mg) and moderate (220 mg) doses of cocoa flavanols reduces platelet aggregation induced by ADP + collagen, as well as by epinephrine + collagen [14]. Catechin and epicatechin, as well as their methylated metabolites, inhibit the formation of A2 thromboxane, a potent vasoconstrictor and platelet aggregator, and they also inhibit ADP-induced aggregation but only at supra-physiological doses [52]. Cocoa attenuates platelet aggregation induced by ADP and thyrombin-receptor activation (thrombin receptor activating peptide SFLLRNamide, TRAP6), but it does not influence the one induced by collagen and thromboxane analogue U46619 [34]. Theobromine, the major methylxanthine in cocoa, also inhibits platelet aggregation induced by ADP and TRAP6, the effects being mediated by PDE inhibition and increase of cAMP [34]. Significant antiplatelet properties of theobromine explain the more complex effects obtained from the use of cocoa products in comparison to those of flavanols alone. It is estimated that the intake of 100 g of dark chocolate with 70% cocoa solids produces an effect on platelets comparable to that induced by a standard dose of aspirin (80 mg), a classic anti-aggregant agent [47]. 

In contrast to the aforementioned data, some studies on the chronic intake of cocoa polyphenols did not find a significant effect on platelet function [68,69]. Ottaviani et al. [31] showed that the daily consumption of up to 2000 mg of cocoa flavanols for 12 weeks did not affect platelet function in humans. Also, a daily intake of up to 1000 mg of cocoa flavanols for two weeks was not associated with changes in platelet function. The authors state that the investigations were performed in healthy subjects and the usual approach to define the health status of participants do not allow a differentiated assessment of the individual health. The divergent or inconsistent results related to the antiplatelet efficacy of cocoa polyphenols could be explained considering the following variables:

(i) Health status/gender of participants. The different basal level of platelet function may result in different responses after the exposure to compounds with antiplatelet potential. In a randomized controlled human intervention trial, Ostertag et al. [70] showed gender-dependent antiplatelet effects of dark chocolate. Acute intake of flavan-3-ol-enriched dark chocolate (907.4 mg flavanols/60 g chocolate) significantly reduces ADP-induced platelet aggregation and P-selectin expression in men and decreases thrombin receptor-activating peptide (TRAP)-induced platelet aggregation in women. The platelets from men are more sensitive to activation via adrenergic and serotoninergic pathways and show more intense thromboxane A2 receptor-related aggregation responses. The women’s platelets have a lower amount of thromboxane A2 receptors, which would explain the inhibitory effect of flavanol-enriched dark chocolate on TRAP-induced aggregation. Also, the use of oral contraceptives and menstrual phase may influence the effects on platelet function. In a critical review on antiplatelet effects of dietary polyphenols, Ostertag et al. [69] noted that the most significant changes on platelet functions have been reported for subjects with single or multiple cardiovascular risk factors. Also, acute intake of dark chocolate (40 g, cocoa > 85%) reduces platelet activation via antioxidant mechanisms only in smokers who have a higher baseline generation of oxidative stress compared to healthy subjects [71];
(ii) Acute or chronic intake. The antiplatelet effects of cocoa flavanols appear to be more intense and meaningful in the case of acute intake. Even a modest amount of flavanols may modulate platelet reactivity in acute studies. The metabolism of cocoa flavanols, bioactivity of metabolites and their persistence could contribute to these findings. The existence of possible different mechanisms for acute and chronic administration does not allow a direct comparison of results from such studies. A better assessment of cocoa flavanols antiplatelet activity should measure both acute and chronic effects in the same study [69];
(iii) Methodology. The assessment of antiplatelet effects of cocoa polyphenols was performed by various experimental approaches that differ in terms of principle, sensitivity, evaluated markers or functions. Besides, the correlations between methods are low. Bleeding time (BT) assesses primary hemostasis by in vivo measurement of bleeding block. Although BT is a simple and quick method, it has the disadvantage that is poorly standardized and is influenced by many variables (skin thickness, temperature among patients). Light transmission platelet aggregometry on platelet-rich plasma (PRP)-LTA is a standard test that evaluates various platelet functions such as platelet activation under action of different agonists (ADP, AA, collagen, and epinephrine, TRAP, thromboxane A2 mimetic U46619) and platelet-to-platelet clump formation in a glycoprotein (GP) IIb/IIIa-dependent manner. The preanalytical conditions (type of anticoagulant, lipid plasma, hemolysis, or low platelet count) as well as procedural conditions (manual sample processing, PRP preparation, use of different concentrations of agonists) may alter the final outcomes [72]. Besides, LTA is a relative non-physiological method, and platelets are not subjected to intense shear conditions [69]. The Platelet Function Analyzer—PFA-100 assesses platelet function in whole blood at the point-of-care under shear stress using collagen (C) plus ADP or collagen plus epinephrine as stimulators of hemostasis. The method presents some limitations such as platelet count-hematocrit-dependence and insensitivity to platelet secretion defects [72]. Platelet analysis based on flow cytometry provides information on platelet functional status in vivo, and includes different methods such as the assessment of platelet activation biomarkers, leukocyte–platelet aggregates or platelet-derived microparticles. However, the preanalytical phase may induce errors, and the measurement of circulating monocyte–platelet is performed under low shear conditions that do not accurately reproduce in vivo processes [69,72];
(iv) Small size of subject lots and different populations [73].

Considering the heterogeneity of studies and the aforementioned aspects, the antiplatelet activity of cocoa polyphenols still remains a topic in debate. Several mechanisms have been suggested but they should be considered from the perspective of previous comments. Also, theobromine may be an important modulator of antiplatelet effects of cocoa products. Such antiplatelet mechanisms of cocoa polyphenols are: (i) modulation of eicosanoids metabolism involved in regulation of vascular homeostasis (stimulation of PGI2 synthesis, inhibition of leukotrienes production) [74,75]; (ii) increase of NO bioavailability, decrease of platelet reactivity [9] and down-regulation of NADPH oxidase and inhibition of platelet isoprostanes [71]; (iii) changes in membrane fluidity [9]; (iv) reduction of the ADP-induced expression of the activated conformation of glycoprotein IIb/IIIa surface proteins and the modulation of platelet integrins that are necessary for platelet-platelet and platelet-endothelial leukocyte interactions [14,76]; (v) inhibition of platelet lipoxygenase; (vi) antagonism of TxA2 platelet receptors [48]; (vii) decrease of phospholipase C activity, an enzyme involved in thrombin-induced platelet activation; (viii) regulation of genes involved in cell adhesion and trans-endothelial migration by acting on the NF-κB and MAPK signaling pathways. These effects are attributed to metabolites of epicatechin and they occur at low, physiological concentrations [52]. 

The well-controlled intervention studies with a large sample size and populations with cardiovascular risk factors along with a uniform methodology are needed to better assess the effects on platelet function exerted by cocoa flavanols.

### 4.5. Modulation of Lipid Profile 

Cocoa polyphenols favorably influence the lipid profile and promote antiaterogenic effects. In vitro studies and studies on cell cultures showed an inhibition of the oxidation of low-density lipoproteins (LDL) and a reduction in the LDL oxidative susceptibility [8]. A diet with various concentrations of cocoa powder (0.5%–10%) or cocoa extract (600 mg/kg per day) for four weeks triggers a reduction in the levels of LDL and triglycerides (TG), a reduction of the LDL oxidability, an increase of the high-density lipoproteins (HDL) and of the plasma antioxidant capacity in normal rats and hypercholesterolemic rabbits [8]. Furthermore, in the case of rabbits with a hypercholesterolemic diet, chronic administration of cocoa proanthocyanidins reduces the levels of plasma lipid hydroperoxides and increases the plasma antioxidant capacity [74]. Numerous clinical trials with varied designs in normocholesterolemic, healthy, mildly hypercholesterolemic subjects, with glucose intolerance or hypertension have demonstrated positive effects on the lipid profile by reducing the plasma levels of LDL, and TG and LDL oxidation, increasing the plasma level of HDL and plasma antioxidant status, reducing the level of several lipid peroxidation markers (TBARS, F2-isoprostanes) [8] and apolipoprotein B (Table 1) [15]. Also, cocoa supplementation may offer protection against postprandial dyslipidemia [23]. Randomized double-blind studies highlighted the HDL enhancing effect of cocoa by the increase of the apolipoprotein A level [52]. The intensity of the effects as well as their profile varies among studies. Some short-term randomized and controlled investigations reported modest changes of the total cholesterol, and LDL, without influencing HDL in patients with cardiovascular risk, and the effects do not seem to be dose-dependent [52]. Other studies indicated that the effects of the cocoa and dark chocolate are higher in patients with cardiovascular risk, in short-term studies and for daily doses of 500 mg polyphenols [77]. The differences between the studies could be explained by the extremely varied designs in terms of the pathophysiologic status of the subjects, the initial lipid profile, the type of cocoa products, the length of the study (2–12 weeks), and the concentration of the polyphenols (187–657 mg proanthocyanidins/day; 46–377 mg epicatechin/day) [15]. At the same time, we must take into account the fact that in the case of cocoa products, other components can also modulate the lipid metabolism. 

The positive effects of the cocoa polyphenols on the lipid profile could involve several mechanisms such as: (i) inhibition of cholesterol absorption in the digestive tract; (ii) inhibition of the hepatic biosynthesis of the cholesterol; (iii) the reduction of the susceptibility of LDL oxidation through changes on their surface [48]; (iv) increase of expression of scavenger receptor B type I, sterol regulatory element binding proteins, ATP binding cassette transporter A1 and apolipoprotein A1 [36].

### 4.6. Anti-Inflammatory Activity

Cardiovascular diseases are currently considered inflammatory diseases; the damage of various cells that participate in cardiovascular functions involves inflammatory and immune responses [12]. Cocoa polyphenols act on several inflammatory mediators and signaling pathways in patients with an increased risk of cardiovascular diseases [74]. On cell cultures, cocoa extracts with 5–100 μg/mL total polyphenols, as well as monomers and oligomeric proanthocyanidins (25 μg/mL) inhibit the production of inflammatory cytokines (IL-1β, IL-2, IL-6, TNF-α), but also the genic expression of iNOS through NF-κB and AP1 pathways. At the same time, in blood mononuclear cells, cocoa polyphenols (monomers to decamers) inhibit the expression of IL-1β. Dose-dependently, they decrease the activity of 15-LOX, 12-LOX and 5-LOX [8]. In mice with a high-fat diet, cacao supplementation (eight percent) for 10 weeks, reduced plasma levels of IL-6 and the TNF-α, IL-6, iNOS and NF-κB expression in adipose tissue. Also, cocoa flavanols can influence other markers of vascular inflammation as soluble adhesion molecules. Thus, the anti-inflammatory effects of cocoa polyphenols contribute not only to the alleviation of endothelial dysfunction, and arterial function and prevention of atherosclerosis, but they also provide benefits in atherothrombotic clinical syndromes. A reduction in mRNA expression of IL-1β, IL-6, E-selectin and vascular cell adhesion molecule (VCAM-1) was also observed in an experimental model of myocarditis in mice [78]. The proanthocyanidins-rich fractions (1–3 μg/mL) and B2 dimer (1.3 μM) reduce the vascular smooth muscle cells inflammatory phenomena by inhibiting the proMMP-2 expression and the MMP-2 activity, an enzyme involved in the degradation of extracellular matrix and the occurrence of atherothrombotic syndromes [78,79]. The intake of flavanol-rich cocoa products (446 mg of flavanols/day) significantly reduces the expression of VCAM-1 in women with postmenopausal hypercholesterolemia. Also, in patients with high cardiovascular risk, the intake of chocolate rich in polyphenols (495 mg of total polyphenols) decreases the levels of intercellular adhesion molecule 1 (ICAM-1). The use of a controlled diet that included 300–900 mg of flavanols/day did not alter ICAM-1 and VCAM-1 levels in obese adults at risk for insulin resistance or in patients with normal/hyper-cholesterolemia. These contradictory data could be explained by different polyphenol intake and extremely diverse pathophysiological status of patients, including the inflammatory picture. Epicatechin and type B proanthocyanidin dimers and their metabolites could be responsible for the effects on soluble adhesion molecules via NF-κB pathway inhibition [78].

### 4.7. Is (−)-Epicatechin the Main Compound Responsible for the Cardioprotective Properties of Cocoa Products?

A very wide range of data from in vitro and animal studies support the cardioprotective potential of (−)-epicatechin. As mentioned in the previous paragraphs, epicatechin has antioxidant properties, alleviates oxidative stress, diminishes ROS-mediated NO inactivation, up-regulates eNOS and increases the bioavailability of NO inducing endothelium-dependent relaxation in animals. Also, it stimulates Nrf2/ARE pathway and inhibits the vascular expression of some proinflammatory and proatherogenic markers (IL-1β, ICAM-1, and TNFα). Epicatechin improves some markers of endothelial function in ApoE knockout mice, a model of atherosclerosis. It reduces plasma ET-1 levels in the apolipoprotein E (ApoE) (−/−) gene-knockout mouse and in Deoxycorticosterone acetate (DOCA)-salt hypertensive rats most probably via Akt-regulation of the ET-1 promoter. The chronic administration of epicatechin prevents the progressive increase in SBP, the proteinuria, and the endothelial dysfunction in uninephrectomized rats chronically exposed to DOCA-salt [80]. In rats after permanent coronary occlusion, the chronic treatment with epicatechin protects against myocardial ischemic injury and preserves left ventricular structure and function [60]. Despite of these promising data, the studies with pure epicatechin in humans are scarce, and the results on endothelial function are conflictual and inconclusive (Table 2). 

Schroeter et al. [87] showed that the oral administration of pure flavanol (−)-epicatechin (1 or 2 mg/kg body weight) to healthy subjects increases FMD and mimics some of acute vascular effects of cocoa, accounting, at least in part, for cocoa beneficial activity. Also, Loke et al. [88] demonstrated that the acute oral treatment with epicatechin (200 mg) modulates some important endothelial markers in healthy subjects. It significantly reduces plasma ET-1 concentration and increases circulating concentrations of vasoactive NO products most probably via eNOs activation and inhibition of NADPH oxidase. On the contrary, Dower et al. [84] did not identify a significant change of FMD or a reduction of BP following acute or chronic epicatechin administration (100 mg/day, four weeks) in apparently healthy older adults but the authors do not exclude the contribution of epicatechin to cardioprotective effects of cocoa. They found that epicatechin negatively modulates fasting plasma glucose and insulin resistance, which are closely related to the endothelial dysfunction. Also, in another interventional study in healthy (pre)hypertensive men and women, they showed that the treatment with epicatechin (100 mg/day, four weeks) causes a decrease of seven percent in sE-selectin, a marker of endothelial dysfunction that is inversely associated with FMD [83]. Barnett et al. [82] showed that a multi-dose intake of epicatechin (50 mg × 2/day, five days) in healthy adults improves mitochondrial enzyme function and increases plasma follistatin levels, an indicator of muscle growth. A careful analysis of the exposed studies reveals several limitations of these interventions, some of them even mentioned by the authors themselves. The main limitations are:

(i) Small number of subjects and statistical underpowered trials [87];
(ii) Large heterogeneity of the study population (40–80 years) and biological variations among subjects [84];
(iii) Dose of epicatechin. As we already noted, EFSA recommends 200 mg of cocoa polyphenols daily for a beneficial effect on endothelial function. Although, Dower et al. [83,84] chosed the dosage of epicatechin in line with the amount of epicatechin present in previous cocoa/chocolate intervention studies (46–107 mg/day); in those studies, the level of total polyphenols was significantly higher (more than 200 mg and even higher than 800 mg). In cocoa products, the effects of epicatechin can be boosted by the pharmacokinetic and pharmacological interactions with other cocoa flavonoids and compounds. The activity of a compound within the natural phytocomplexes may be different as intensity or even sense from that of pure compound. The interactions between compounds in the phytocomplex affect their solubility, bioavailability or bioactivity and lead to a nuanced expression of biological response. In fact, even the authors mentioned that the dose of 100 mg epicatechin is likely to be too low to exert an effect on NO metabolism, the main target of vasodilatory mechanisms. Moreover, using a nonlinear meta-regression model with a Bayesian approach, Ellinger et al. (2012) [88] showed that the dose of ingested epicatechin influences the mean treatment effect. In this respect, authors showed that the daily intake of 25 mg epicatechin via cocoa consumption (but not as pure compound) can reduce BP through an increased availability of NO. In the study of Schroeter et al. [87], the dosage of epicatechin can be even lower or higher than 100 mg, depending on the body weight of subjects. In this context, the occurrence of the effects at low doses is difficult to explain. Maybe the fact that the subjects are more age-homogenous (25–32 years) exerts a positive influence;
(iv) Health status of subjects. Baseline values of cardiovascular status and metabolism are different for young adults (25–32 years) and older adults (over 50 years), the last category being included in the studies of Dower et al. [83,84]. The type of treatment with epicatechin (acute vs. acute-on-chronic effect) may influence the final outcome. Dower et al. [84] showed that it is possible to reach a plateau regarding the effect on the endothelial function after 4 weeks of epicatechin administration, and one additional acute dose could not elicit more effect on FMD. The present data do not allow to define the cardiovascular profile of epicatechin in humans. The topic remains in discussion, and further long-term well-designed studies, with a larger number of subjects and appropriate methodology, are needed to gain more insight into the cardioprotective potential of epicatechin.

## 5. Safety of Cocoa Polyphenols

Daily intake of cocoa flavanols in amounts up to 2000 mg was well tolerated in healthy adults; only mild gastrointestinal effects have been reported [31]. Cocoa polyphenols may reduce iron absorption. A cocoa beverage with 100–400 mg of polyphenols per serving could decrease iron absorption by about 70% [89]. Also, the administration of cocoa products with other caffeine-containing foods and drinks may enhance the side effects of caffeine.

## 6. Limitations of Cocoa Studies 

Many of the current clinical studies using cocoa products show some shortcomings that should be corrected to allow a better assessment of cocoa’s cardioprotective effects. The major critical points are: (i) a large heterogeneity in terms of cocoa products type (cocoa powder, dark chocolate, milk chocolate, cocoa beverages, semi-sweet chocolate baking bits), dose of flavanols, control, pathophysiological status and age of subjects; (ii) human intervention trials with a small number of subjects; (iii) few cross-over designed studies; (iv) lack of data related to polyphenolic profile of cocoa products (type and proportions of polyphenols as monomers, oligomers and polymers); (v) short-term studies (less than two weeks) and poor controls [8,58]; (vi) lack of data regarding plasma polyphenol concentrations [8]; (vii) numerous studies including only healthy subjects; (viii) lack of randomized controlled trials concerning the effects of cocoa on pivotal cardiovascular outcomes as cardiovascular mortality, myocardial infarction or stroke [49]. Therefore, the design of future clinical studies should comply with the following guidelines: (i) randomized, controlled, cross-over, multi-dose trials [10]; (ii) long-term trials in larger cohortes [34]; (iii) analytically well-characterized and standardized cocoa products in terms of flavanol and procyanidin content and profile and specification of the concentrations of fats, sugars, milk proteins [2,10,16]; (iv) flavanol-free controls [2]; (v) well-characterized participant population (life-style and diet habits, medical status) and the inclusion of subjects with elevated cardiovascular risk factors [16]; (vi) quantification of circulating flavanol levels and assessment of characteristic flavanol metabolites [16]; (vii) use of a dose of cocoa product, which could be included in the daily diet [10]. 

## 7. Future Perspectives

Future research on cardiovascular effects of cocoa polyphenols should consider the following topics: (i) clinical trials on individual polyphenols of cocoa: flavanol-monomers and proanthocyanidins [52]; (ii) assessment of cardiovascular activity of conjugates metabolites of cocoa polyphenols (mainly epicatechin metabolites) [2]; (iii) identification of the minimal doses of cocoa/chocolate that need to be ingested to exert cardioprotective effects [49]; (iv) investigation of molecular mechanisms of cocoa flavanols [52]; (v) investigation of polyphenol bioavailability from different cocoa-containing matrices [10].

## 8. Conclusions

Cocoa and cocoa polyphenols appear to exert promising cardioprotective effects in humans. Their clinical use depends largely on the clarification of the issues related to the pharmacokinetic and pharmacological properties as well as the interactions between polyphenols and other compounds of cocoa in well-designed studies.

## Figures and Tables

**Table 1:** Outcomes of recent (2014–2016) clinical trials of cocoa products.

Year	Study Design	No. of Participants	Aprox. Mean Age (years)	Sex	Comorbidities	Cocoa Product Intervention	TF (mg/day)	EC (mg/day)	Duration (weeks)	Outcomes	Ref.
2016	Randomized, double-blind, placebo-controlled, crossover	32	70	M + F	Chronic heart failure (stable on GDMT)	Dark chocolate	1064 (HFDC); 88 (LFDC)	ns	4	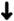 Plasma NT-proBNP (HFDC) vs. LFDC and baseline; 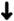 DBP vs. LFDC and baseline; ♦ No change in platelet function.	De Palma et al. [19]
2016	Randomized, placebo controlled crossover	20	62	M	None	Dark chocolate	770	150	1 dose	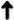 FMD vs. control; 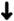 AIx vs. control.	Dower et al. [20]
2016	Randomized, placebo-controlled, double-blind	57	65	M + F	End-stage renal disease with chronic hemodialysis (pharmacological medication)	Cocoa powder	900	ns	4	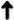 FMD vs. control; 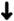 DBP vs. control.	Rassaf et al. [21]
2015	ENRICA cohort study	1272	18–65 and over	M + F	Coronary heart disease, Hypertension, Diabetes, Hypercholesterolemia, Stroke	Chocolate	ns	ns	3 years	No evidence of beneficial effects of chocolate regular consumption on physical or mental components of *Health-related Quality of Life*	Balboa-Castillo et al. [22]
2015	Randomized, double-blind, crossover, postprandial study	18	ns	M + F	Type 2 diabetes, Obese	Cocoa beverage	480	40	1 dose	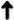 HDLc vs. placebo; 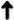 Serum insulin concentration vs. placebo; 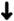 Large artery elasticity vs. placebo; ♦ No effect on total cholesterol, LDLc, TG, glucose, hsCRP vs. placebo; ♦ No changes in SBP, DBP vs. placebo.	Basu et al. [23]
2015	Randomized, controlled, double-blind, crossover	20	18–70	ns	None	Cocoa powder	80–800	17–168	1	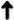 FMD vs. control; 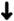 SBP, DBP, PWV, ET1 vs. control	Grassi et al. [24]
2015	Blinded, randomized, controlled, crossover	21	ns	ns	Symptomatic peripheral artery disease	Dark chocolate	ns	ns	1 dose	♦ No effect on microvascular function vs. cocoa-free control chocolate.	Hammer et al. [25]
2015	Randomized, double-blind, controlled, parallel-group	22 young 20 elderly	22/60	M	None	Cocoa powder	900	128	2	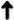 FMD, arteriolar and microvascular vasodilator capacity, red blood cell deformability vs. baseline; 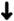 PWV, total peripheral resistance vs. baseline.	Heiss et al. [26]
2015	Randomized, controlled, blind open-label	79	42	M + F	Hypertension (grades I and II) treated with captopril or telmisartan	Dark chocolate + dehydrated red apples + green tea	425.8	ns	24	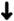 SBP, DBP, TG, hsCRP vs. control	De Jesús Romero-Prado et al. [27]
2015	Randomized, controlled, crossover	22	33–64	M + F	Mild hypertension	Dark chocolate	602.70	ns	8	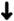 DBP, SBP vs. baseline; ♦ Improvement of subjects status from hypertensive to normotensive; ♦ No significant changes in CAVI vs. baseline; ♦ No significant changes in lipid profile, blood glucose, blood insulin vs. baseline.	Koli et al. [28]
2015	Randomized, placebo-controlled, double-blind, parallel	40	18–43	ns	None	Cocoa extract	250	ns	1 dose; 4	♦ No significant changes in peripheral and central blood pressure vs. baseline; ♦ No significant changes in central blood flow vs. baseline.	Massee et al. [29]
2015	Randomized, double-blind	24	22	F	None/Overweight Obesity	Cocoa powder	640	48	4	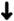 Haptoglobin, total and proinflammatory monocyte CD62L expression in obese women vs. baseline; 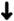 Endothelial microparticles in obese and overweight women vs. baseline.	McFarlin et al. [30]
2015	Randomized, parallel-arm, double-masked, controlled dietary intervention	46	35–55	M + F	None	Food-grade cocoa extract	2000	220	12	♦ No significant in BP, platelet function, liver panel (albumin, bilirubin, ALT, AST, AlkPhos), metabolic markers (glucose, hemoglobin, hematocrit, urea, Na^+^, K^+^, Ca^2+^, Cl^-^, leucocytes, erythrocytes) vs. cocoa flavanols-free control	Ottaviani et al. [31]
2015	Double-blind, randomized, crossover	15	18–35	M	None	Cocoa powder	1.4–10.9 /kg	0.37–1.5 /kg	1 dose	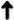 FMD vs. baseline	Rodriguez-Mateos et al. [32]
2015	Randomized, placebo-controlled, double-blind	60	57	M + F	Type 2 diabetes and Hypertension	Dark chocolate	450	ns	8	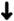 FBG, SBP, DBP, hsCRP, apolipoprotein B vs. baseline; 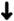 FBG, SBP, DBP in dark chocolate group vs. control.	Rostami et al. [33]
2015	Randomized, double-blind, placebo-controlled	32	45–70	M	Pre-hypertension, Mild hypertension	Dark chocolate	1064 (HFDC); 88 (LFDC)	ns	6	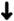 Platelet aggregation induced by thrombin-receptor activation, ADP vs. baseline; 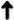 Heart rate (LFDC) vs. baseline; ♦No changes in total cholesterol, LDLc, HDLc, TG vs. Baseline ♦No significant changes in SBP, DBP (HFDC ) vs. baseline or LFDC.	Rull et al. [34]
2015	Randomized, double-blind, controlled	100	44	M + F	None	Cocoa powder	450	64	4	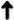 FMD, HDLc vs. control; 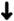 SBP, DPB, PWV, total and LDLc vs. control.	Sansone et al. [35]
2015	Two consecutive controlled, crossover	44	29	M + F	None/Mild hypercholesterolemia	Cocoa powder rich in dietary fibers/polyphenols	43.8/45.3	9.3/18.9	4	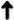 HDLc after the 2nd intervention vs. baseline; 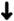 Plasma glucose and IL-1β in cocoa product rich in dietary fiber group vs. baseline.	Sarriá et al. [36]
2015	Randomized, double-blind, crossover	7	24–31	M	None	Pure flavanols (monomers and polymers)	1 mg/kg bw (EC, PCB1); 2 mg/kg bw PPC	-	Single dose	Glucuronidated, sulfated, methylated (-)EC and DHPV are predominant metabolites in blood and urine.	Wiese et al. [37]
2014	Randomised, double-blind, crossover	40	64/27/60	M + F	None (young and old)/Coronary artery disease	Cocoa beverage	375	59	4	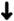 CD144^+^ EMP, CD31^+^/412^−^ vs. baseline	Horn et al. [38]
2014	Randomised, parallel and double-blind	50	57	M + F	None	Cocoa powder	414.26	153.44	4	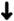 Oxidized LDLc vs. control and vs. baseline; 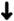 MPO, ICAM1 vs. baseline.	Ibero-Baibar et al. [39]
2014	Non-randomized, controlled, crossover	44	29	M + F	None/ Hypercholesterolemia	Cocoa powder	45.3	18.9	4	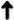 HDLc vs. baseline; 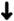 IL 10 vs. baseline.	Martinez-López et al. [40]
2014	Double-blind, controlled, parallel-arm	90	61–85	M + F	Diabetes, Hypercholesterolemia Hypertension (pharmacological treatments), Former smokers	Cocoa drinks	993 (HF); 520 (IF); 48 (LF)	185 (HF); 95 (IF); 5 (LF)	8	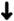 SBP, DBP (HF, IF) vs. LF; 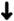 Plasma glucose concentrations (HF, IF) vs. LF; 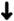 Insulin resistance (HF, IF) vs. LF; 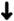 LDLc, TG, total cholesterol vs. LF; 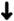 Plasma total 8-iso-prostaglandin F2α (HF, LF) vs. LF; 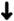 Circulating insulin (HF, IF) vs. LF; 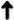 HDLc (HF, IF) vs. LF.	Mastroiacovo et al. [41]
2014	Randomized, controlled, crossover	44	29	M + F	None/Hypercholeste-romia	Cocoa powder	44.1	9.3	4	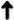 HDLc vs. baseline; 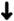 Glucose, IL-1β and IL-10 vs. baseline.	Sarría et al. [42]
2014	Randomized, single-blinded, prospective placebo-controlled	60	65	ns	None/Glaucoma	Dark chocolate	ns	ns	1 dose	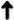 Mean dilatation of the retinal venules in age-matched controls, but not in glaucoma patients; 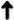 Venous vasodilatation in control group, but not in the glaucoma group.	Terai et al. [43]
2014	Randomized, double-blind, 2 period, crossover	30	52	M + F	Overweight, Moderate obesity	Dark chocolate and sugar-free cocoa powder	814	73.6	4	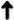 Basal blood flow, basal and peak diameter of the brachial artery, vs. baseline	West et al. [44]

Abbreviations: Aix, augmentation index; ALT, alanine aminotransferase; AST, aspartate aminotransferase; AlkPhos, alkaline phosphatase; CAVI, cardio-ankle vascular index; CHD, chronic heart disease; DBP, diastolic blood pressure; DHPV, 5-(3’,4’-dihydroxyphenyl)-valerolactone; EC, epicatechin; EMP, endothelial microparticles; ET1, endothelin 1; F, females; FBG, fasting blood glucose; FMD, flow-mediated dilation; GDMT, guideline-directed medical therapy; HDLc, high density lipoprotein-cholesterol; HFDC, high-flavanol dark chocolate; HF, high flavanol intake; hsCRP, highly sensitive C-reactive protein; ICAM-1, intercellular adhesion molecule 1; IL, interleukin; IT, intermediate flavanol intake; LDLc, low density lipoprotein-cholesterol; LF, low flavanol intake; LFDC, low-flavanol dark chocolate; M, males; MPO, myeloperoxidase; NF-κB, nuclear factor kappa-light-chain-enhancer of activated B cells; ns, not specified; NT-proBNP, N-terminal pro-B-type natriuretic peptide; PCB1, procyanidin B1; PPC, pure polymeric procyanidin fraction from cocoa; PWV, pulse wave velocity; SPB, systolic blood pressure; TF, total flavanols.

**Table 2:** Outcomes of clinical trials of epicatechin (2016–2006).

Year	Study Design	No. of Participants	Aprox. Mean Age (years)	Sex	Comorbidities	Form of EC Intervention	EC (mg/day)	Duration (weeks)	Outcomes	Ref.
2016	Randomized, multicenter, placebo-controlled, double-blind	30	18–55	M + F	Hypertriglyceridemia	Capsules	100	4	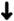 TG, TG/HDLc ratio, hsCRP vs. baseline	Gutiérrez-Salméan et al. [81]
2015	Non-randomized open label	9 (single-dose); 8 (multiple-dose)	23–68 (single-dose); 22–45 (multiple-dose)	ns	None	Dissolved in water	50; 100	Single-dose; multiple-dose, 5 days	↑ Plasma nitrite vs. baseline; ↑ Platelet mitochondria complexes I, IV, citrate synthase activities (multi-dose) vs. baseline; ↑ Plasma follistatin levels (multi-dose) vs. baseline.	Barnett et. al. [82]
2015	Randomized, double blind, placebo-controlled crossover	35	40–80	M + F	Pre-hypertension	Capsules	100	4	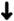 Plasma soluble endothelial selectin vs. control	Dower et al. [83]
2015	Randomized, double-blind, placebo-controlled, crossover	33	40–80	M + F	None	Capsules	100	4	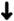 Fasting plasma insulin and insulin resistance vs. control	Dower et al. [84]
2014	Pilot, open-labeled, crossover	20	28	M + F	None/Overweight	Capsules	1 mg/kg	1 dose	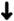 Blood glucose and TG after 2 h vs. control	Gutiérrez-Salméan et al. [85]
2008	Randomized, placebo-controlled, crossover	12	43	M	None	Dissolved in water	200	1 dose	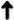 Plasma S-nitrosothiols and nitrite after 2 h and urinary nitrate after 5 h vs. Baseline 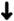 Plasma entothelin-1 after 2 h vs. baseline	Loke et al. [86]
2006	Randomized, double-blind, crossover	6 (3 + 3)	25–32	M	None	Dissolved in water	1 or 2 mg/kg	1 dose	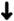 FMD and PAT responses after 2 h vs. baseline and vs. control	Schroeter et al. [87]

Abbreviations: EC, epicatechin; F, females; FMD, flow-mediated dilation; HDLc, high density lipoprotein-cholesterol; hsCRP, highly sensitive C-reactive protein; M, males; ns, not specified; PAT, peripheral arterial tonometry; TG, triglycerides.
